# Quiet eye training–based intervention can ameliorate inhibitory control but not visuospatial working memory in children with ADHD

**DOI:** 10.1002/brb3.3251

**Published:** 2023-09-15

**Authors:** Rudolf Psotta, Javad Sarvestan, Ludvík Valtr, Ondrej Ješina

**Affiliations:** ^1^ Faculty of Physical Culture Palacky University Olomouc Czech Republic; ^2^ College of Physical Education and Sport PALESTRA Prague Czech Republic

**Keywords:** ADHD, attention, inhibition, quiet eye, training, working memory

## Abstract

**Intro:**

The purpose of this study was to investigate the effects of quiet eye training (QET) on inhibitory control, visuospatial working memory (WM), and tonic attention in children with attention‐deficit hyperactivity disorder (ADHD).

**Methods:**

Forty‐eight children with ADHD aged 9–12 years were randomly assigned to QET and control (CON) groups. The QET group practiced targeted hand–eye tasks within a QET protocol developed to optimize controlled attention and gaze through eye fixations. We used the go/no‐go (GNG) test, the Corsi test, and the reaction test of alertness (RTA) to verify the effects of QET on inhibition control, WM, and tonic attention.

**Results:**

QET group showed significantly shorter reaction times, a higher number of correct responses, and a lower number of omissions in the GNG inhibition test after QET as compared to the pre‐measurements, whereas the CON group did not demonstrate significant changes in this test. The measures of WM (Corsi test) and tonic attention (RTA) did not change significantly with the QET‐based intervention.

**Conclusion:**

The study demonstrated that the QET protocol, which includes instructions and a video demonstration to optimize eye fixation on a target during aiming tasks, is acceptable and usable for children with ADHD. Overall, a short‐term, 5‐week visuomotor training intervention based on the quiet eye paradigm was shown to be effective in improving inhibitory control and focused visual attention, but not visuospatial WM and intrinsic attention in 9–12‐year‐old children with inattentive or combined ADHD.

## Graphical abstract

The purpose of this study was to investigate the effects of quiet eye training (QET) on inhibitory control, visuospatial working memory (WM), and tonic attention in children with attention‐deficit hyperactivity disorder (ADHD). QET group showed significantly shorter reaction times, a higher number of correct responses, and a lower number of omissions in the go/no‐go inhibition test after QET as compared to the pre‐measurements, whereas the CON group did not demonstrate significant changes in this test.

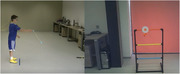

## 1 INTRODUCTION

Behavioral inhibition and working memory (WM) are considered primary executive functions, which underpin the core features of attention‐deficit hyperactivity disorder (ADHD), such as inattention, hyperactivity, and impulsivity (Baddeley, 2007; Tarle et al., 2019). According to the factor model of executive functioning, inattention and inhibition in a continuous performance task and WM are three dimensions of the executive functioning (Barkley et al., 2001). WM covers processes of coding, temporary storage, active maintenance, updating, and the manipulation of information for a few seconds for the purpose of performing tasks (Baddeley, 2007; Siegelbaum & Kandel, 2013). Although the functional connection of WM to attention has been explicitly investigated by only a few studies to date, attention is considered to be embedded in WM (Cowan, 2005). Researchers assume that the central executive component of WM is an attentional controller that is responsible for supervising and coordinating the storage and rehearsal systems, focusing attention, and protecting temporarily stored information from competing distracting information (Alderson et al., 2017; Baddeley, 2007). Attention mechanisms are also responsible for the visual information processed into WM (Knudsen, 2007), and errors in visuospatial perception caused by impaired attention result in poor‐quality input of information going into WM (Carelli et al., 2022).

The second cognitive function of interest—inhibitory control (response inhibition)—is the cognitive process of suppressing or withholding unrelated or dominant motor responses (Skogan et al., 2014). This depends on the finishing of go and stop processes that are initiated with the presentation of prepotent go‐stimuli and stop‐signals, respectively (Logan et al., 1997; Tarle et al., 2019). There is evidence that inattention symptoms such as impaired focused attention and sustained attention to visual tasks in children with ADHD are associated not only with a prolonged reaction time (RT) and higher variability of RT to relevant stimuli but also with poorer inhibition indicated by a higher commission error rate (responses to irrelevant stimuli) in the inhibition tasks when compared to typically developing (TD) children (Alderson et al., 2017; Epstein, 2012; Skogan et al., 2014; Tarle et al., 2019). The theoretical view on this linkage of inhibition deficits and impaired attention is supported by the finding of a significant prediction of inattentive symptoms in children with ADHD by inhibition, together with WM (Tarle et al., 2019).

Attention is reflected in abnormalities of gaze control during tasks demanding visual attention based on the bidirectional functional linkage of attention and gaze control (Kleberg et al., 2020; Vickers, 2009). There is strong evidence that visual information that reaches the fovea via eye fixation is subsequently highly prioritized for further cortical processing (Raz & Buhle, 2006), and any shift in gaze to a new location is always preceded by a shift in attention (Vickers, 2009). As visual attention is a substantial mechanism for gaze control (Vickers, 2009), eye movements and fixations during goal‐directed actions are used as oculomotor markers of attention (Sanchez et al., 2020; Türkan, 2016; Vickers, 2007) and inhibition control (Bucci et al., 2017; Fried et al., 2014; Huang & Chan, 2020; Kleberg et al., 2020). Indeed, compared to their TD peers, children with ADHD display atypical eye movement patterns, such as difficulties in maintaining eye fixations, saccadic control (Bucci et al., 2017; Türkan, 2016), including the inhibition of automatic saccades (Huang & Chan, 2020), and the suppression of eye blinks (Fried et al., 2014) in tasks demanding fixations or anti‐saccade tasks.

Based on functional linkages between visual attention and gaze control, quiet eye training (QET) has been developed (Vickers, 2007). This visuomotor training consists of performing a targeting, interceptive, or other visuomotor task during which the individual is provided with specific instructions and video demonstrations of a skilled model of eye fixations in order to optimize his/her controlled attention and gaze in turn refining visuomotor control (Miles et al., 2017; Vickers, 2007; Vine et al., 2014). The paradigm of QET is based on the findings that final eye fixation on a relevant object before the initiation of a targeting or interceptive body movement, called “quiet eye,” has been shown to be indicative of superior visuomotor control (Miles et al., 2015; Vickers, 2007; Vine et al., 2014). The explanation for this is that longer and earlier quiet eye processed into the central nervous system is critical for cognitive processes (Vine & Wilson, 2011) and the motor pre‐programming of an action (Vickers, 2007).

There is considerable support for the hypothesis that an intervention based on the QET principle could be effective for the amelioration of inhibitory control and visuospatial WM. First, attention underpinned by inhibition and embedded in WM processes (see above) can be ameliorated by QET because it provides explicit stimuli for controlled attention and gaze via the performance of goal‐directed motor tasks, as shown in studies with children (Miles et al., 2015; Wood et al., 2017). It was presumed that QET‐based intervention would have the effect of improving inhibitory control, due to the finding of a longer quiet eye duration during the execution of a targeting task that individuals performed in conditions in which an internal distractor, induced by experimental manipulation of cognitive anxiety, was present (Moore et al., 2012; Vine & Wilson, 2011). A similar type of cognitive training on eye fixation demonstrated that attention and inhibition capacities in children with neurodevelopmental disorders can be improved by explicit support for the control of observing stimuli or events (Carrelli et al., 2022; Lai et al., 2020). In addition, gaze‐contingent eye tracking training has been implemented to enable children with executing complex cognitive tasks to improve sustained attention, inhibition, and WM (for a review, see Carelli et al., 2022).

To our knowledge, the effectiveness of QET‐based intervention on inhibitory control and WM in children with ADHD has not yet been examined. Therefore, the aim of the study was to investigate whether this type of attention‐focused training via the performance of targeting tasks can be beneficial for enhancing inhibitory control and visuospatial WM. Based on current knowledge that inhibitory control and WM processes are functionally associated with attention that is also interconnected with gaze control, we hypothesized that the QET‐based intervention can ameliorate inhibitory control and visuospatial WM in children with ADHD.

## 2 METHODS

### 2.1 Study design and participants

The study was designed as a randomized double‐blind controlled trial. Forty‐eight children were recruited from 11 mainstream schools. The children met two inclusive criteria—the age of 9–12 years and the diagnostic criteria for the inattentive and combined type of ADHD according to DSM‐5 (American Psychiatric Association [APA], 2013). The exclusive criteria were severe anxiety, disruptive mood dysregulation disorder, behavioral disorder, intellectual disorder, other psychotic disorders, visual and hearing impairment, physical and neurological impairments affecting body mobility, and no correction of gaze. The children were diagnosed with ADHD before entering the study, made by licensed psychologists at the Educational‐Psychological Advice Centre, and based on information on developmental history, medical reports, interviews with parents, teachers, and a child, cognitive testing, and observation.

The participants were randomly divided into two groups. The QET group, *n* = 26, age 10.3 ± 1.1 (4 females), comprised 21 children with ADHD‐I and 5 children with ADHD‐C. Two of the children from the QET group were left‐handed. The control (CON) group, *n* = 22, age 10.6 ± 1.0 (5 females) comprised 18 children with ADHD‐I and 4 children with ADHD‐C; three of the group were left‐handed. The parents reported the administration of stimulant medication (Ritalin or Concerta) in nine children (18.9%, five in the QET group) to manage their ADHD, and no child took other psychiatric medications. No significant differences were observed between the groups in terms of age (*p* = .209), sex (*p* = .364), dominant hand (*p* = .347), ADHD subtype (*p* = .597), and medicated children (*p* = .131). A priori power analysis showed that 48 participants would be sufficient to identify a significant effect of the 2 independent variables (pre‐ vs. post‐measures, QET vs. CON group) using a factorial 2 × 2 mixed‐effects ANOVA, with a power (1 − *β*) of .90, effect size *f* of .25, and an *α* of .05.

Written informed consent from the legal guardians of all the children we obtained to participate in the intervention program focused on reducing attention problems. Ethical approval for the study was obtained from the Institutional Review Board (code 31/2018). The participating children, their legal guardians, the testers, and the instructors of the QET‐based intervention were not aware of the aim of the study.

Each participant attended a pre‐measurement (first week) and post‐measurement (seventh week). These measurements incorporated three neuropsychological tests (see below). From the second to the sixth week, the QET group underwent QET, one lesson per week, that is, a total of five lessons. The individuals in the CON group did not participate in any specific training/intervention on cognitive/executive functions during the 6‐week pre–post interval.

### 2.2 Pre‐ and post‐measurements

The pre‐ and post‐measurements included three computer‐based neuropsychological tests, carried out in one session from 8.30 to 11.00 a.m. in a quiet room, and in counterbalanced order in each group. Each participant performed the tests in comfortable sitting position on a chair at a table, with his/her elbows/arms resting on the table. The height of the chair was adjusted for each child so that the knee angle was approx. 90°. The participants viewed the screen from a distance of approx. 52 cm. The tests were performed with the use of a laptop computer with a screen of 15 in. and the resolution of 1920 × 1080 pixels.

At the beginning of each test, the tester provided the participant with very brief instructions on the test task and procedure. The participant then obtained the computer‐driven instructions and performed practice trials of the test (see below). When it was obvious that he/she understood the task, the test followed.

### 2.3 Neuropsychological assessment

#### 2.3.1 Go/no‐go (GNG) inhibition test

The go/no‐go (GNG) PsyToolkit test (https://www.psytoolkit.org/cgi‐bin/psy2.5.4/login) was used to assess inhibitory control. The task for the participant was to respond as quickly as possible to a go‐stimulus (green oval, width 5 cm, height 2 cm) by pressing the spacebar and to refrain from this response in the case of no‐go‐stimulus (red oval, equal dimensions as the green oval). The GNG stimuli were presented individually at the center of a black background of a computer screen for a duration of 500 ms at an interstimulus interval of 2.000–4.000 s, at a ratio of GNG stimuli (go/stop trials) of 80/20, presented at random. The participant was initially presented with 20 practice stimuli, and then, he/she completed 100 trials. The time duration of the test, including the instruction and practice, is 10 min.

The following variables were assessed as measures of inhibition: mean RT to go‐stimuli (GNG MRT), intraindividual coefficient of variability of RT to go‐stimuli calculated as SD of RT/MRT.100 (GNG ICV RT), percentage of correct responses across GNG trials (accuracy), omission errors—no response given during go‐trials (GNG omission), commission errors—response given during no‐go‐trials (GNG commission). Only correct responses to go‐stimuli with RT ≥ 100 ms were included in the calculation of MRT and ICV RT. RT < 100 ms in no‐go‐trials was not used for the calculation of the number of commission errors and percent accuracy.

A GNG simple RT task has been proven to measure children's response inhibition and sustained attention (Li et al., 2016; Tarle et al., 2019). Moderate‐to‐high reliability of the type of GNG inhibition test has been reported (Tyburski et al., 2021; Weafer et al., 2013). For the assessment of the results of the GNG tests, estimations of reference range were used for the variables that were calculated in our pilot study with TD children aged 10–11 years (*n* = 62) according to the formula 95% CI = mean ± *t*_0.975,_ *_n_*_−1_·√(*n* + 1)/*n*·SD. Reference ranges were as follows: for MRT 367–431 ms, number of omission 3–15 and commission 0–6.

#### 2.3.2 The Corsi test of working memory

The Corsi forward block‐tapping test for children (Corsi test) (Schuhfried, 2011a) was used to assess visuospatial short‐term WM. The task for the participant was to reproduce presented sequences from two to eight blocks with the aim of remembering it and pointing to as many blocks as possible. On the screen, the participant sees nine irregularly positioned blocks. A mouse cursor in the shape of a hand moves about the screen and points to a block, which briefly lights up. After the block has lit up, the “hand” points to another block, and to another, and so on. This presentation of a particular sequence concludes with a signal tone. The participant then tries to tap with a mouse on the same blocks in same order in which the “hand” pointed. Each sequence with a discrete number of blocks is presented in three trials. After these trials, the number of blocks increases by one. The test finishes as soon as a participant performs three successive trials incorrectly. The test also terminates automatically after the third sequence of eight blocks, that is, after 21 trials. Two practice trials precede the test trials. The total duration of the test ranged from 4 to 9 min in our sample.

The following four variables were used for analyses: immediate block span (IBS)—the longest sequence that the participant correctly reproduced in at least two of three trials presented; IBS is a measure of short‐term visuospatial memory span; the number of correctly reproduced sequences (CorrSeq); the number of incorrectly reproduced sequences (IncorrSeq); sequencing errors (SeqErr)—the number of sequences for which the positions of all the blocks in a sequence were correctly tapped but the order in which this was done was incorrect.

Good internal consistency of IBS was reported as *r* = .760 (Schuhfried, 2011a). The Corsi forward test is valid for the assessment of visuospatial WM span (Baddeley, 2021; Schuhfried, 2011a), and it reflects the development of cognitive functions and demonstrates close correlations with more complex cognitive functions (Schuhfried, 2011a).

#### 2.3.3 Reaction test of alertness

To assess tonic (intrinsic) attention and arousal regulation the reaction test of alertness (RTA), version 31 of the Vienna Test System (VTS) (Schuhfried, 2011b) was used. The test is a simple RT task that consists of 28 reactions to a target stimulus (yellow circle, diameter 3 cm) displayed in the center of a black background of a computer screen. From the basic position of the index finger of the dominant hand on the home button (a diameter of 2 cm), the participant responded to the target stimulus as quickly as possible by pressing the rectangular black response button (5.5 × 1.5 cm^2^) on the operating panel. After the response, the participant placed his/her index finger back on the home button to adopt the basic position. RT is measured as the interval between the moment of appearance of the target stimulus and the moment the index finger left the home button. The display of the target stimulus remained until a response was detected up to a maximum of 1000 ms. The stimuli were presented in interstimulus intervals from 2500 to 6500 ms. Before the test trials, the participant responded to 10 target stimuli. The total duration of the test was 8 min.

The following variables were assessed: MRT as a measure of tonic attention/arousal regulation (MRT‐ton), intraindividual coefficient of variation of RT (ICV RT; %) as a measure of the intraindividual variability of arousal regulation, and a number of omission errors. The following incorrect responses were identified by VTS software and not included in calculations of the variables: (i) concurrent pressing of the home button and the response button with two different fingers; (ii) use of a finger other than the index finger to press the response button, indicated by an interval of <50 ms between taking the index finger off the home button and pressing the response button; (iii) taking the finger off the home button before the appearance of the stimulus. Excellent reliability, *r* = .965, for RT in the RTA was reported (Schuhfried, 2011b).

### 2.4 Training phase, training protocol

**FIGURE 1:**
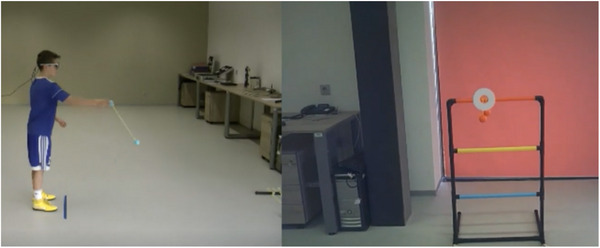
The split‐screen video of the throwing bolas task (the motor video on left half and the gaze video on right half of screen).

The QET‐based intervention for the QET group consisted in performing the targeting tasks (Table 1) accompanied with observation of a split‐screen video with footage of gaze and body movement during a throwing action of a skilled model, supplemented with the instructor's instructions to emphasize the focusing of their gaze on a target. The split‐screen video for each task was created from eye‐tracker recording (gaze video on right half of screen), including the location of a focal point within the visual field with a target, and from bodily movement records taken from the sagittal view of the performer (motor video on left half of screen) during the performance of a targeting task by a very skilled 12‐year‐old individual (Figure 1). The gaze and motor videos were synchronized in the split‐screen video for the given task. The split‐screen videos were created at playback speeds of 100%, 50%, and 25%.

**TABLE 1:** Targeting tasks and their modifications for the quiet eye training–based intervention.

Training session	The major tasks
First	Throwing the ball against a wall (2 m distance) and catching with two hands after bouncing
Second	Overarm throwing a beanbag on one of three vertical openings of the construction (2 m distance)
Third	Dart throwing from distance 2.37 m
Fourth	Beanbag underarm throwing on one of three horizontal openings of the construction (2 m distance)
Fifth	Throwing a bolas tool on a vertical rung
	**Modifications of the tasks**
	Different distances from a target
	The change of a target of alternative ones (with exception of first session)
	Changes of angle of the position of the participant to a target (with exception of first session)
	Using non‐preferred hand for throwing
	Different frequencies of the changes of the modifications mentioned above
	Different combinations of the modifications mentioned above

The QET consisted of five training sessions, one session per week, with 7 days between two consecutive sessions. The training sessions always took place at the school attended by the given participant, in a sufficiently large room and under the guidance of a trained postgraduate instructor. At each training session, one targeting task and modifications thereof were practiced. The sessions were run according to the QET protocol for a throwing task described by Miles et al. (2015), with the exception of no instructions concerning the technique of throwing but the use of split‐screen videos at three different playback speeds: Step 1: Instruction and actual demonstration of the task by the instructor to the participant. Step 2: The participant watched the split‐screen video on a tablet with focusing of gaze on the motor video at a playback speed of 100%, 50%, and 25% first (observation approx. 90 s) and then on the gaze video at a playback speed of 100%, 50%, and 25% (approx. 90 s). The participant's observation of the gaze video was accompanied with instructions to support the optimizing of gaze before the initiation of a throwing arm movement and during the throw (observation of the scene; early gaze at the target or point on the wall in the task before the initiation of the throw; maintaining gaze on the target during and after the initiation of the throw; attempting to reduce eye movement; maintaining of open eyes). Step 3: The participant was asked to summarize how to gaze in the pre‐throw phase and throw phase, in order to check his/her understanding of how to adapt visual attention to the target (short review, questioning). Step 4: The participant performed 30 practice trials of a task; after each 5 trials, the instructor encouraged the participant to focus on a target in order to execute a targeting task. Step 5: The participant watched the gaze video, accompanied with brief instructions on the major points for optimizing gaze while throwing (see Step 2). Step 6: The participant performed the final 20 practice trials of the task.

The participant was allowed a short break at any step during the training protocol if needed. After a short 3–4 min break, the participant performed various modifications of the task with the use of the six‐step training protocol presented above. The total time of each lesson was 35 min. The same training protocol was used for all lessons. The sessions took place every week on the same day ± 1 day.

### 2.5 Data analysis

In order to verify the normality of the metric variables that were incorporated into the mixed linear model, the Shapiro–Wilk test and the Kolmogorov–Smirnov test (*α* = .05) were used. The zero‐hypothesis concerning the normality of residues was rejected only for the variable of ICV RT%. This abnormality was caused by a number of insufficiently remote observations. However, the histogram indicated that this did not constitute a systematic deviation from normality, and that the division of the residues converged toward a normal division.

The results of the variables of a metric type were analyzed using a factorial 2 × 2 mixed‐effects ANOVA with a fixed factor of Group (QET, CON) and Time (pretest, posttest). The *F*‐test with adjusted degrees of freedom (df) based on the Satterthwaite approximation was used. Subject factor (48 levels) was treated as a random effect. To analyze the effects of Group and Time factors on the variables that are rather of categorical type (numbers), a generalized mixed linear model was used. For this model, we used the *F*‐test with df based on the residual method. The effect size of interactions was quantified using partial eta squared, with an interpretation of *ηp* ^2^ = .01, .06, and .14 as small, medium, and large effects, respectively. The effects of time in the QET and CON groups were quantified using the following formula:

$$d\;=\;\frac{M_{post}-M_{pre}}{\sqrt{\sigma_{\varepsilon }^{2}+\sigma_{\tau }^{2}}}$$

in which *M* indicates group mean, $\sigma_{\varepsilon }^{2}$ is the residual variance of the model, and $\sigma_{\tau }^{2}$ is the variance of the proband random factor. The residual variance of the model is a variance of the proband random factor, and *d* can be interpreted analogously to Cohen's *d*, *d* = .20, .50, and .80 as low, medium, and large effects, respectively. The level of *α* = .05 was set for all tests. All data analyses were conducted with IBM SPSS Statistics (Version 28; IBM).

## 3 RESULTS

Descriptive statistics are presented in Table 2. MRT in the GNG test (GNG MRT) in both groups was within the limits of reference range (RR), whereas the number of GNG omissions and GNG commissions were above the upper limits of RR (see Section 2.3.1). In the QET group, a significantly shorter GNG MRT time was determined in the post‐measurements, as well as a higher number of correct responses (accuracy) and a lower number of GNG omissions in comparison with the pre‐measurements, and variability of RT (GNG ICV RT%) was reduced with marginal significance (Table 3). In the CON group, no significant changes were determined in the results of the GNG test upon a comparison of the pre‐ and post‐values. The interactions of Group × Time were significant for GNG omission and marginally significant for accuracy. Neither the Time factor for both groups nor the interactions of Group × Time were significant for the number of commissions (Table 3).

**TABLE 2:** Means and standard deviations in quiet eye training (QET) group and control group.

	QET group		Control group	
Variable	Pretest	Posttest	Pretest	Posttest
***Reaction test of alertness (RTA)***				
MRT (ms)	370 ± 47	355 ± 47	352 ± 67	368 ± 57
ICV RT (%)	16.8 ± 5.0	5.3 ± 4.5	16.2 ± 5.4	15.8 ± 4.3
RTA omission error (*n*)	0.7 ± 0.7	0.5 ± 0.8	0.3 ± 0.6	0.8 ± 1.6
***Go/no‐go test***				
GNG MRT (ms)	415 ± 20	405 ± 25	402 ± 26	400 ± 21
GNG ICV RT (%)	11.8 ± 2.5	12.8 ± 2.7	12.6 ± 3.0	13.2 ± 3.0
Accuracy (%)	59.5 ± 13.6	66.3 ± 16.0	68.3 ± 11.4	68.6 ± 10.0
GNG omission errors (*n*)	36.1 ± 14.2	30.0 ± 16.4	20.4 ± 11.0	24.1 ± 9.9
GNG commission errors (*n*)	4.4 ± 3.0	3.6 ± 2.4	11.1 ± 9.7	7.3 ± 3.5
***Corsi test***				
Work memory span IBS (*n*)	3.6 ± 1.0	3.9 ± 1.0	4.0 ± 1.3	4.3 ± 1.3
CorrSeq (*n*)	4.9 ± 2.3	5.3 ± 2.5	5.8 ± 3.4	6.6 ± 3.1
***IncorrSeq (n)***				
	4.3 ± 1.2	4.3 ± 1.0	4.3 ± 1.4	4.3 ± 1.4
SeqError (*n*)				
	1.1 ± 0.8	1.4 ± 1.1	2.0 ± 1.2	1.9 ± 1.0

Abbreviations: CorrSeq, the number of correctly reproduced sequences; GNG ICV RT, intraindividual coefficient of variability of reaction times to go‐stimuli; GNG MRT, mean reaction time to go‐stimuli; IBS, immediate block span; ICV RT, intraindividual coefficient of variation of reaction times; IncorrSeq, the number of incorrectly reproduced sequences; MRT, mean reaction time; QET group, quiet eye training group; SeqError, the number of sequencing errors.

**TABLE 3:** Comparison of effects in quiet eye training (QET) group and control group.

Variable	Effect in QET group			Effect in control group			Interaction Group × Time		
	*d*	*t*(48)	*p*	*d*	*t*(48)	*p*	*η^2^*	*F*(1,48)	*p*
***Reaction test of alertness (RTA)***									
MRT (ms)	−.27	−1.426	.161	.28	1.336	.188	.004	3.797	.057
ICV RT (%)	−.30	−1.242	.221	−.08	−.303	.763	.004	.381	.540
RTA omission (*n*)	−.10	.563	.574	.44	−2.440	.017	.053	5.124	.026
***Go/no‐go test***									
GNG MRT (ms)	−.42	−2.502	.016	−.04	−.248	.805	.024	2.285	.138
GNG ICV RT (%)	.37	1.780	.082	.21	.946	.349	.003	.259	.613
Accuracy (%)	.03	2.972	.005	.51	.145	.885	.038	3.629	.063
GNG omission errors (*n*)	−.44	−2.787	.008	.27	1.582	.125	.092	9.310	.004
GNG commission errors (*n*)	−.15	−.583	.563	−.71	−2.566	.014	.024	2.231	.142
***Corsi test***									
Working memory span (*n*)	.61	−1.339	.184	.72	−1.389	.168	.000	.005	.944
CorrSeq (*n*)	.49	−.890	.376	.85	−1.302	.196	.000	.074	.786
IncorrSeq (*n*)	.07	−.109	.913	.06	−.120	.905	.000	.000	.988
SeqError	.27	−.968	.336	−.16	.442	.674	.011	1.015	.316

*Note*: *d*, effect size; *t, F*, test criteria; *η* ^2^, partial eta squared.

Abbreviations: CorrSeq, the number of correctly reproduced sequences; GNG ICV RT, intraindividual coefficient of variability of reaction times to go‐stimuli; GNG MRT, mean reaction time to go‐stimuli; ICV RT, intraindividual coefficient of variation of reaction times; IncorrSeq, the number of incorrectly reproduced sequences; MRT, mean reaction time; *p*, a probability level; QET group, quiet eye training group; SeqError, the number of sequencing errors.

The pre‐values of the main variable in the Corsi test—IBS (Table 2) in the QET and CON groups—were below average, corresponding on average to the 17.1st percentile and the 24.4th percentile, respectively, according to the norms. The variables of the Corsi test did not show a significant difference between the pre‐ and post‐values in any of the groups. At the same time, no Group × Time interaction was significant for any variable of the Corsi test (Table 3).

In the pre‐measurement of RTA, the QET and CON groups scored significantly below average MRTs, which corresponded to the 9.8th and 14.3th percentiles, respectively, according to the norms (Table 2). After the end of the training, the QET group achieved a lower MRT in contrast with the CON group, but these differences in the pre‐ and posttest were not significant. Nonetheless, the Group × Time interaction was demonstrated to be marginally significant (Table 3). Changes in the variability of RT ICV RT% in the post‐ and pretest were not significant in any group (Table 3). The number of omissions was significantly reduced in the posttest in the QET group, in contrast with the insignificant difference of post‐ versus pre‐values in the CON group. At the same time, the Group × Time interaction for the number of omissions was significant (Table 3).

## 4 DISCUSSION

In comparison with the regular population of the same age, the QET and CON groups on average had a delayed response to the visual stimulus in the RTA test, impaired control of response inhibition in the GNG task, and a below average scope of visuospatial WM in the Corsi test. These findings supported the association of inattention behavior with underlying cognitive processes in the participants, in a line of diagnostic features of ADHD outlined by DSM‐5 (APA, 2013) and findings of previous research (e.g., Alderson et al., 2017; Skogan et al., 2014; Tarle et al., 2019).

This study demonstrated that QET in 9–12‐year‐old children with ADHD can bring about an improvement of inhibition control, but not of visuospatial WM. The individuals in the QET group demonstrated a shortening of MRT, a reduction of variability of RT, and a reduction of omission errors in the GNG task after the end of the training. These metrics of GNG inhibition are considered indexes of sustained attention or controlled‐focused attention that are commonly associated with attention and vigilance deficits (Sjöwall et al., 2013; Tarle et al., 2019; Vuontela et al., 2013; Wright et al., 2014). According to the information processing theory (Schmidt et al., 2011MRT achieved), in a GNG simple reaction task reflects the total time needed for three processes—stimulus detection and recognition (differentiation of the green oval from the red oval and vice versa), and neural programming of motor response during go‐trials. In accordance with this theory, the shortening of MRT in the QET group may reflect more rapid processing of visual information as a consequence of better maintenance of focused attention. This suggestion is also supported by the findings of more significant continuous performance task (CPT) inattention in adolescents with ADHD while no difference in WM and CPT inhibition as compared to the control group (Barkley et al., 2001).

However, at the same time, QET did not lead to an improvement of intrinsic (tonic) alertness assessed in the simple RTA task. RT measured in this type of the reaction task indicates rather difficulties in basic information processing rather than sustained attention (Moreno‐García et al., 2015; Sjöwall et al., 2013). In accordance with this theory of information processing (Schmidt et al., 2011), in contrast with the GNG task, RT in the RTA task does not incorporate the process of differentiation of the stimulus. Furthermore, in addition to this process, the response to go‐stimuli is associated with specific cognitive processes. It is possible that the planning of go‐responses may be prolonged due to a competition between a prepotent response tendency triggered by predominant go‐stimulus presentations and a tendency to increase the response threshold to reduce the probability of responding to a no‐go‐stimulus (Verbruggen & Logan, 2008). It is probable that the GNG task places greater cognitive demands than the simple RT task and will thus be associated with a higher activation of top–down controlled attention, whereas the generation of responses in a simple RT may take place by means of uncontrolled cognitive processes. A difference between the cognitive processes incorporated in the GNG task and the RTA task is indicated by the 12.2% and 14.1% longer RT in the GNG task than RT in the RTA in the QET and CON groups, respectively, and the statistically significant difference of RT in these two tasks (*p* < .001). It therefore appears that QET‐based intervention may effectively stimulate controlled focused attention more than intrinsic attention in individuals with ADHD.

The observed reduction of omission errors in the QET group may have been contributed to the more effective differentiation of go‐stimuli from no‐go stimuli, and the more rapid generation of motor responses. An omission error was identified when an individual failed to respond to a go‐stimulus within 500 ms, similarly as in the GNG tasks used on children with ADHD in previous studies (e.g., Epstein et al., 2012; Li et al., 2016). The shortening of the time required for processing information to less than 500 ms in the go‐trials may explain the reduction of omission errors. Previous research indicated an improvement of resistance to the disruption of attention following 3‐day QET. It determined more effective control of attention, indicated by the behavior of the eyes during the performance of a targeting task under the conditions of internal distractors associated with the manipulated induction of cognitive anxiety (Moore et al., 2012; Vine & Wilson, 2011). Our study suggests that a reduction of omission errors and an acceleration of responses to go‐stimuli in the QET group may have been mediated by improved focused attention, stimulated during the practicing of targeting tasks in the QET‐based intervention.

In addition to the above‐discussed changes of the metrics of GNG inhibition, at the same time, there was a reduction of commission errors in the QET group. Commission error is used as the index of inhibitory control (Verbruggen & Logan, 2008; Wright et al., 2014). The study by Tarle et al. (2019) suggests that commission errors reflect an individual's inhibitory response ability, rather than a strong activation of prepotent response. One of the hypotheses concerning cognitive mechanisms of inhibition is that the go and stop processes take place independently of one another, and if the stop process finishes before the go process, response inhibition is successful and no response is emitted, whereas when the go process finishes before the stop process, response inhibition is unsuccessful (Verbruggen & Logan, 2008). It is not possible to say what specific cognitive mechanisms associated with inhibition may be influenced by QET, which placed demands on focused attention. Nevertheless, the timely termination of the stop process could be supported by the quicker differentiation of the no‐go‐stimulus from the go‐stimulus as a consequence of improved focused attention stimulated by QET. Improvement of focused attention is supported by the findings of a positive change of further GNG inhibition markers, specifically MRT and omission errors.

QET‐based intervention did not lead to an improvement of WM. Our assumption of the effect of training on WM was based on the embedded‐process model of WM (Cowan, 2005) and the multicomponent model, in which the central executive component of WM is the attention controller (Baddeley, 2021, 2007) (see Section 1). One of the possible explanations of the ineffectiveness of QET on visuospatial WM may be the different visuo‐cognitive processes in the performed targeting tasks and in the memory performance of the Corsi test. Performance of targeting tasks in QET was combined with an explicit teaching of effective eye behavior, and thereby an optimization of focused attention (Miles et al., 2015). Repeated stimulation of eye fixation on a target may have supported the individual's receipt of an external focusing of attention, which also facilitates visuomotor coupling (Wulf & Lewthwaite, 2016). In comparison with this, visuospatial WM evidently combines external and internal focusing of attention, where external attention refers to selection through perception and internal attention refers to selection from memory (Hitch et al., 2018). Attention in the Corsi forward task is drawn to spatial rehearsal function, including the visual perception of sequential spatial information that needs to be stored (Baddeley, 2007; Schuhfried, 2011a). More specifically, spatial rehearsal in this kind of WM task requires a conscious, attention‐based shift from one remembered location and its corresponding stimulus (a block) to the next one (Schuhfried, 2011a), in contrast with visual “one‐cue” focused attention during a targeting task. In addition, the Corsi task involves the reproduction of this kind of visuospatial information, which also requires internal attention (Hitch et al., 2018). It could be concluded that although the tasks performed in the QET‐based intervention required episodes of attention focused on a single external goal, the Corsi task is associated with maintaining attention on spatial locations, storage and retrieval of the visuospatial information with a probable combination of externally and internally focused attention.

The study was first investigation focused on the potential effect of the QET intervention on cognitive functions in children in ADHD. Therefore, we took this investigation as the pilot study to reveal whether the intervention based on the QET could possibly affect two selected cognitive functions in children. To implement this training into clinical practice, next research is needed to evaluate potential changes of clinical symptoms of ADHD. Another potential limitation for the generalization of the results of the study could be the mixed sample of children with ADHD‐I and ADHD‐C. Children with **ADHD‐**C might exhibit more impulsivity in gaze control (Luo et al., 2022), pronounced disinhibition, more behavioral problems, emotional instability, and anxiety, whereas deficits in sustained attention and vigilance during visual tasks are more pronounced in ADHD‐I (Rostami et al., 2022; Sanchez et al., 2020). This specific feature of cognitive and behavioral functioning may have influenced the effectiveness of visual attention training. However, the proportion of children with a diagnosis of ADHD‐C out of the total number in the group was very small, specifically 19.2% in the QET group and 18.2% in the CON group, without any statistically significant difference between the two groups. Furthermore, the proportion of children with medication was marginal, and the QET and CON groups did not differ in the proportion of medicated children. Nonetheless, the data about medication were dependent upon the response of the children's parents. However, the baseline neuropsychological tests confirmed that both groups on average manifested an abnormal level of inhibition, WM, and intrinsic attention. The validity of the results of the study may also have been influenced by the size of the cohort with reference to the used statistical model. Nonetheless, an a priori power analysis showed that 48 participants would be sufficient to identify a significant effect for the used statistical model with a power (1 − *β*) of .80, effect size *f* of .36, and an *α* of .05.

## 5 CONCLUSIONS

Overall, short‐term, 5‐week visuomotor training intervention based on the QE paradigm proved to be effective in improving inhibitory control and visually focused attention, but not visuospatial WM or basic, intrinsic attention in 9–12‐year‐old children with inattentive or combined type of ADHD. The probable reason for the ineffectiveness of QET on visuospatial WM is that this training stimulates other aspects of attention associated with fixation of the eyes on a target in a frontal visual field, in comparison with attention control of sequenced perception of visuospatial stimuli and cognitive processes of WM. The study demonstrated that the QET protocol, which includes instructions and video demonstration for the purpose of optimizing eye fixation on a target during the performance of targeting tasks, is acceptable and usable for children with ADHD. In psychological or educational practice, performing visuomotor tasks accompanied with the instructions that would encourage a longer and/or earlier fixation of eyes on the target or moving object could be useful to stimulate visual attention and the ability to suppress responses to unrelated or disturbing stimuli in children with ADHD. However, future research is needed to investigate the possible effects of the QET‐based intervention on a broader set of clinical symptoms of ADHD.

## CONFLICT OF INTEREST STATEMENT

The authors named immediately above confirm that they have no affiliation or involvement with any organization or entity with any financial interest (such as honoraria; educational grants; participation in speakers, membership, employment, consulting, stock ownership or other ownership interest, and professional testimony or patent‐licensing arrangements) or nonfinancial interest (such as personal or professional relationships, affiliations, knowledge, or beliefs) in the subject matter or materials discussed in this manuscript.

### PEER REVIEW

The peer review history for this article is available at https://publons.com/publon/10.1002/brb3.3251.

## Data Availability

Data are available on request due to privacy/ethical restriction.
